# A Walking Case of Typhoid Fever

**Published:** 1899-12

**Authors:** J. H. Reuss

**Affiliations:** Cuero, Texas


For Texas Medical Journal.

### A Walking Case of Typhoid Fever.—Perforation and Death.*

BY J. H. REUSS, M. D., CUERO, TEXAS.

D. M. S.; male; age, 26; married; broom manufacturer. Was called in haste to see this patient July 25, as he was suddenly stricken with severe pain in the abdomen. When first seen patient presented the symptoms of a severe abdominal affection; features pinched, pulse 104, temperature 102, respiration rapid, and pain very severe over region of the bladder; had not voided urine in twenty-four hours, except half ounce or so. Had taken a cathartic pill in the morning, and after a few hours an enema which caused three large actions.

Upon passing catheter two ounces of clear urine was drawn. Warm applications were made over the abdomen, calomel one-fourth grain with one grain extract hyoscyamus was ordered every hour. One-fourth grain morphine hypodermically not having relieved pain, another one-fourth grain was given in half an hour. In the night, as the pain was insufferable, another one-fourth grain of

*Read at annual meeting of the West Texas Medical Association, San Antonio, October 26, 1899.

morphine hypodermically was given, also chloroform inhalations with very little effect, a complete anæsthesia of chloroform was his only relief. During this whole period of time abdomen was much distended and tympanitic.

July 26, 7 a. m., entire lower abdomen was much tumefied and on palpation dull, much pain as before, and bladder very sensitive. Upon passing catheter one-half pint of urine was drawn, after which urine passed freely.

Diagnosis, general peritonitis with effusion. Operation was decided upon, but refused by patient; suffering continuing.

Patient had been ailing for three or four weeks, but kept at his work, and had been moving heavy machinery for several days. Much emaciation and loss of strength. A few hours before he was taken with this severe pain he was setting up a heavy stove and, as he says, felt something snap, followed by a pain in the abdomen.

On the morning of July 27, symptoms very grave; pulse 142, hardly perceptible, respiration irregular and very rapid, and although believed to be in a moribund condition a laparotomy was done under protest at the urgent request of the family.

A large incision was made in the median line, and upon opening the peritoneum a large quantity of very offensive fecal matter poured out.

General peritoneal cavity was filled with the same and plastic adhesions. After a rapid cleansing of the cavity, upon close inspection of the bowels, a circular perforation the size of a pea was detected in ileum, from which fecal matter was running. This was sutured and the peritoneal cavity was washed out with saline solution. During the whole time of anæsthesia pulse was imperceptible, and hardly a beat of the heart could be detected, notwithstanding several hypodermics of strychnine and whiskey. Patient was put to bed and expired about one and one-half hours afterwards. Autopsy was refused by the family.

From previous history of the case and the occurrence of the perforation, patient had no doubt, so to say, a "walking case" of ulcerated patches in the bowels, and notwithstanding his constantly increasing emaciation and loss of strength, he continued his work. Not until he was lifting the stove did the bowels perforate, and the very severe symptoms appear.

The danger of such cases, as well as the earnest necessity of rest in the bed, is, I think, in this case well demonstrated. Had a physician been in attendance in due time the result would surely have been different.

